# Effects of Calcitonin-Gene-Related-Peptide on Auditory Nerve Activity

**DOI:** 10.3389/fcell.2021.752963

**Published:** 2021-11-12

**Authors:** Colleen G. Le Prell, Larry F. Hughes, David F. Dolan, Sanford C. Bledsoe

**Affiliations:** ^1^ Department of Otolaryngology, University of Michigan, Ann Arbor, MI, United States; ^2^ Department of Speech, Language, and Hearing, University of Texas at Dallas, Richardson, TX, United States; ^3^ Department of Surgery, Southern Illinois University School of Medicine, Springfield, IL, United States

**Keywords:** cochlea, CGRP, auditory nerve, auditory brainstem response, lateral olivocochlear efferent

## Abstract

Calcitonin-gene-related peptide (CGRP) is a lateral olivocochlear (LOC) efferent neurotransmitter. Depression of sound-driven auditory brainstem response amplitude in CGRP-null mice suggests the potential for endogenous CGRP release to upregulate spontaneous and/or sound-driven auditory nerve (AN) activity. We chronically infused CGRP into the guinea pig cochlea and evaluated changes in AN activity as well as outer hair cell (OHC) function. The amplitude of both round window noise (a measure of ensemble spontaneous activity) and the synchronous whole-nerve response to sound (compound action potential, CAP) were enhanced. Lack of change in both onset adaptation and steady state amplitude of sound-evoked distortion product otoacoustic emission (DPOAE) responses indicated CGRP had no effect on OHCs, suggesting the origin of the observed changes was neural. Combined with results from the CGRP-null mice, these results appear to confirm that endogenous CGRP enhances auditory nerve activity when released by the LOC neurons. However, infusion of the CGRP receptor antagonist CGRP (8–37) did not reliably influence spontaneous or sound-driven AN activity, or OHC function, results that contrast with the decreased ABR amplitude measured in CGRP-null mice.

## Introduction

A variety of evidence suggests a role for Calcitonin-gene-related-peptide (CGRP) in auditory function, with CGRP released by the lateral olivocochlear (LOC) efferent neurons mediating the neural response to sound. The guinea pig is a common model for studies of the LOC system. LOC efferents have been immunolabeled with antibodies to CGRP in the cochlea in guinea pigs (Takeda et al., 1987; Sliwinska-Kowalska et al., 1989; Ylikoski et al., 1989; Ohno et al., 1993; Cabanillas and Luebke, 2002). Other studies of the LOC system document CGRP-like immunoreactivity in additional species including mice (Maison et al., 2003a; Wu et al., 2018), rats (Kitajiri et al., 1985; Takeda et al., 1986; Lu et al., 1987; Tohyama et al., 1989; Ylikoski et al., 1989; Kuriyama et al., 1990; Merchan-Perez et al., 1990), hamsters (Simmons et al., 1996), cats (Lu et al., 1987), and humans (Kong et al., 2002).

Immunolabeling of the human cochlea revealed CGRP-like immunoreactivity in the inner spiral bundle and tunnel spiral bundle suggesting expression of CGRP in both the LOC and medial olivococlear (MOC) efferent neurons with no notable differences from base to apex (Schrott-Fischer et al., 2007). CGRP immunoreactive terminals are distributed under the inner hair cells (IHCs) in approximately equal numbers throughout the length of the cochlea in rats (Vetter et al., 1991) and mice (Maison et al., 2003a) although some mouse strains may have a gradient with less robust CGRP presence in the apex (Wu et al., 2018). There are some reports that CGRP-positive labeling of the LOC system is robust across the length of the guinea pig cochlea (Sliwinska-Kowalska et al., 1989; Ylikoski et al., 1989) although there are also reports that CGRP-positive cells are less abundant in the apex and upper third turn of the guinea pig cochlea (Safieddine and Eybalin, 1992). Sliwinska-Kowalska et al. (1989) investigated CGRP distribution in pigmented female guinea pigs weighing 150–300 g, Ylikoski et al. (1989) used 200–350 G guinea pigs but did not specify sex or strain, and Safieddine and Eybalin (1992) used pigmented guinea pigs weighing 200–250 g with sex not specified, thus it is not known if there are sex or strain (pigmented/albino) differences in CGRP distribution. In the only study assessing potential sex differences in the effects of CGRP within the LOC system, Allen and Luebke (2017) reported no functional differences between male and female CGRP-null mice. The lack of sex differences in that mouse study contrasts with sex differences in CGRP immunoreactivity observed in other tissues from rat and from human (Valdemarsson et al., 1990; Herbison and Spratt, 1995; Ji et al., 2019), with sex differences in humans potentially explaining sex differences in the efficacy of antimigraine medications that target CGRP receptors (Barbanti et al., 2021; de Vries Lentsch et al., 2021).

The mature CGRP receptor is a G-protein coupled receptor (GPCR) composed of three proteins, including calcitonin-like receptor (CLR), receptor activity-modifying protein (RAMP1), and CGRP-receptor component protein (RCP) (for additional detail, see Dickerson et al., 2016). Post-natal maturation of the CGRP receptor has been documented in the mouse (Dickerson et al., 2016). In mice, the CGRP receptor complex within the cochlea shows developmental maturation over the first 3-months (Dickerson et al., 2016). The guinea pig is precocial, whereas the mouse is altricial, and receptor development may follow different timelines in guinea pig and mouse. Post-natal receptor maturation of the CGRP receptor has not been studied in the guinea pig. Interestingly, observations of CGRP expression in the Type II SGNs have also been reported, although release of CGRP from type II afferent terminals in the cochlear nucleus has not been established and functional actions are not yet determined (Wu et al., 2018; Vyas et al., 2019).

The lateral superior olive (LSO) is the site of origin for the descending LOC efferent neurons. CGRP-like immunolabeling of the LSO is also well described in multiple species (Lu et al., 1987; Vetter et al., 1991; Simmons and Raji-Kubba, 1993; Safieddine et al., 1997; Simmons et al., 1997; Moore et al., 1999; Reuss et al., 1999). Co-localization of CGRP and other LOC transmitters has been described by several groups, and species differences have emerged with respect to colocalization as well as overall distribution. For example, in the human LSO, Moore et al. (1999) describe CGRP-positive terminals that are also positive for antibodies to choline acetyltransferase (ChAT); these cells were distinct from a second population which immunostained for ChAT but not CGRP. In the rat LSO and cochlea, Vetter et al. (1991) describe CGRP-positive terminals that are also positive for antibodies to ChAT; these cells were distinct from a second population which immunostained with antibodies to glutamic acid decarboxylase (GAD, the enzyme that decarboxylates glutamate to make GABA). In contrast, in the mouse, GABA and CGRP were extensively co-localized with ACh in LOC terminals (Maison et al., 2003a), whereas dopaminergic (DA) neurons were a separate subpopulation (Darrow et al., 2006). In studies evaluating CGRP co-localization with other LOC transmitters in guinea pigs, LSO cell bodies have been found to co-localize CGRP and encephalin (enk) (Tohyama et al., 1990), or CGRP, enk, and ACh (Safieddine and Eybalin, 1992). Finally, extensive colocalization has been observed with double and triple immunolabeling for ChAT and either CGRP, enk, GAD, or tyrosine hydroxylase (TH, the enzyme responsible for catalyzing the conversion of L-tyrosine to the DA precursor dihydroxyphenylalanine) (Safieddine et al., 1997).

That CGRP is present in the LSO and the LOC terminals in a variety of species suggests the potential for a functional role of CGRP in modulating the ascending auditory signal. This possibility has been explored, and supported, by data from a small number of studies. Depressed amplitude of auditory brainstem response (ABR) wave I in αCGRP-null mice (Maison et al., 2003b) has been shown, an effect that is consistent with excitatory modulation of sound-evoked auditory nerve (AN) activity by CGRP. One interpretation is that sound stimulation drives release of CGRP by LOC efferents during acoustic stimulation of the LOC reflex loop, and in the absence of CGRP release (in the αCGRP-null mice), sound-evoked responses are depressed. Alternatively, if CGRP is tonically released, the observed effects might also be explained by changes in AN spontaneous response properties. In mammals, AN fibers with higher spontaneous firing rates have lower (better) thresholds, are less sharply tuned, and adapt to sound more rapidly than other fibers with lower spontaneous firing rates (see Sachs and Abbas, 1974; Liberman, 1978; Rhode and Smith, 1985; Winter et al., 1990; Muller and Robertson, 1991; Yates, 1991). Consistent with the hypothesis that CGRP can increase spontaneous neural activity, application of CGRP produced an increase in spontaneous neural activity in the *Xenopus* lateral line organ (Adams et al., 1987; Sewell and Starr, 1991; Bailey and Swell, 2000a; Bailey and Swell, 2000b).

In the study by Maison et al. (2003b), which revealed that ABR amplitude was depressed in αCGRP-null mice, there were no corresponding changes in ABR threshold, distortion product otoacoustic emission (DPOAE) threshold and amplitude, or the strength of the MOC reflex. Allen and Luebke (2017) similarly report intact DPOAE responses in αCGRP-null mice. The lack of change in DPOAE outcomes [reflecting no change in outer hair cell (OHC) function] and the MOC metric are perhaps somewhat surprising. Earlier literature described CGRP-containing nerve endings on OHCs in rodents (Cabanillas and Luebke, 2002; Maison et al., 2003a) and humans (Kong et al., 2002; Schrott-Fischer et al., 2007), suggesting CGRP may function as an MOC transmitter as well as an LOC transmitter (see also Vyas et al., 2019). The limited DPOAE changes detected in the knock-out mice might provide an incomplete description of the role of CGRP in auditory physiology, however, if there is central nervous system compensation during development (as suggested by May et al., 2002, who evaluated auditory sensitivity in mice lacking alpha-9 receptors). In another recent investigation, mature CGRP-null mice (3 months of age) not only had reduced cochlear nerve activity, they were also observed to have increased thresholds both in quiet and in broadband noise background (with thresholds measured using pre-pulse inhibition of the acoustic startle reflex) (Allen and Luebke, 2017). Development of deficits from 1 to 3 months of age was consistent with the timing of CGRP receptor maturation in mice shown by Dickerson et al. (2016).

In contrast to earlier research using CGRP-null mice, the current study evaluated the effects of infusing CGRP into the cochlea on auditory function, including assessments for both LOC and MOC-like functional effects. Effects of CGRP on sound-evoked response were evaluated using both CGRP and CGRP fragment 8–37, which acts as an antagonist at CGRP receptors. To evaluate the hypothesis that CGRP mediates changes in spontaneous AN activity as well as sound-driven AN activity, we measured electrical activity at the round window membrane, which provides a measure of ensemble spontaneous activity of the AN fiber population (Kiang et al., 1976; Dolan et al., 1990; Groff and Liberman, 2003). Changes in DPOAE amplitude and onset adaptation were used to assess effects of the study drugs on OHC responses. The use of experimental designs that combine both agonists and antagonists has allowed the effects of exogenous drug delivery and tonic endogenous release to be distinguished for the LOC transmitter DA (Garrett et al., 2011); the current data extend understanding of putative LOC transmitter substances to the peptide CGRP.

## Methods

### Animal Model

Fourteen guinea pigs (Elm Hill Breeding Labs, Chelmsford, MA) were used in these experiments (5 female, 5 male, sex records not available for 4 animals). Two additional animals were excluded from the investigation based on permanent profound post-operative hearing loss. Animal weights were ∼250–300 g on arrival (approximately 4 weeks old) and ∼350–500 g (approximately 6 weeks old) at study entry. All animals were maintained with free access to food (Guinea Pig Chow, PMI Nutrition International Inc., Brentwood, MO) and water. The animal care program was AALAC accredited. Husbandry met or exceeded all applicable standards, including the Guide for the Use and Care of Laboratory Animals, prepared by the National Research Council (1996). The University Committee on Use and Care of Animals at the University of Michigan approved all animal care and testing protocols.

### Apparatus and Procedures

#### Surgical Procedure

The studies described here used a chronic unilateral left-ear drug-infusion paradigm. CGRP has a molecular weight of ∼3,800; it is a large enough molecule that it is unlikely to readily diffuse across the round window membrane (as larger molecules less readily diffuse across this membrane, see Goycoolea and Lundman, 1997; Mikulec et al., 2008). Therefore, it was delivered directly into the cochlea using a surgically implanted cannula. The surgical implant procedures were closely modeled after those we have described previously (Le Prell et al., 2004). Animals were anesthetized (40 mg/kg ketamine, 10 mg/kg xylazine), then a cannula filled with an artificial perilymph (AP) solution (145 mM NaCl, 2.7 mM KCl, 2.0 mM MgSO_4_, 1.2 mM CaCl_2_, 5.0 mM HEPES; pH = 7.40, osmolality = 280–285 mOsm) was inserted through the wall of the left cochlea via a small fenestra slightly lateral to the round window. The site of insertion was at a place with a best frequency of approximately 22.4 kHz (based on surgically-induced threshold deficits described in Le Prell et al., 2004). A silastic ball located 0.5 mm away from the end of the cannula prevented over-insertion of the cannula and prevented leaking of fluids from the cochlea. The other end of the cannula was connected to an osmotic mini-pump (model 2002, 0.5 μl/h x 14 days; Durect corporation, Cupertino, CA), also filled with the AP solution.

After inserting the cannula, a ball electrode (0.25 mm diameter, constructed of teflon-coated platinum-iridium wire) was carefully placed on the left round window membrane following procedures we have described previously (Le Prell et al., 2006). A ground wire was inserted into the middle ear via the defect in the bulla, and carboxylate cement (Durelon, ESPE, Germany) was used to seal the bulla defect and permanently fix both the cannula and the electrodes in place. The opposing ends of the electrodes were soldered to a two-pin connector (HSS-132-G2, Samtec Inc., IN) prior to the onset of the surgical procedure. Methyl methacrylate cement (Jet Repair Acrylic, Lang Dental Manufacturing, IL) was used to fix the cannula and the connector for the electrodes to the skull, and to seal the tissue edges surrounding the head-mounted connector. The post-auricular incision was then sutured and the incision cleaned.

AP was infused unilaterally for 12–14 days during which a variety of post-operative baseline data were collected. Guinea pigs then underwent a brief surgical procedure during which a small incision exposed the depleted osmotic pump, the depleted pump was extruded, the cannula clamped and cut, and a new osmotic-pump attached. The new pump was filled with either 200 μM CGRP (CGRP rat, Sigma Chemical C0292, CAS No. 96827-03-1; dissolved in AP) (*n* = 5; four female, sex not recorded for one animal) or 250 μM CGRP fragment 8–37 (synthetic human CGRP fragment 8–37, Sigma Chemical C2806, CAS No. 119911-68-1; dissolved in AP) (*n* = 7; four male, one female, sex not recorded for two animals).

#### Electrophysiology

Animals were anesthetized (20 mg/kg ketamine, 5 mg/kg xylazine), and placed on a warm heating pad to maintain body temperature. Acoustic stimuli were brief pure-tone stimuli (4, 8, 16, or 22.4 kHz) presented at levels ranging from 0 to 100-dB SPL in 5-dB increments (5-msec duration, 0.5-msec rise-fall; 10/sec). Acoustic stimuli were generated using Tucker-Davis Technology (TDT; Alachua, FL) System II/System III hardware and SigGen32 software. Signals were converted to analog (DA1), filtered (FT6-2, F_c_ = 40 kHz), attenuated (PA5), and presented using a 200-Ohm transducer (Beyer Dynamic, Farmingdale, NY) coupled to the animals’ ear canal *via* vinyl tubing. Cochlear potentials were filtered (300–3,000 Hz) and amplified (1,000x) using a Grass P55 amplifier. BioSig32 (TDT) was used to average 25 evoked responses for each frequency/level combination. Threshold to evoke a compound action potential (CAP) response of the auditory nerve was defined as the sound level that produced a 10-μV response; threshold estimates were determined using linear interpolation. CAP responses were measured using the implanted electrode; thus, testing was unilateral. CAP testing was repeated at 2–3 day intervals to monitor stability of post-surgical responses during AP infusion (the control condition), with additional testing at 2–3 day intervals during CGRP or CGRP (8–37) infusion (the experimental conditions) to monitor progressive changes as the experimental substances increased in concentration in the cochlea with continued infusion.

#### Round Window Noise

Baseline tests were conducted once during the second week of unilateral AP infusion (between days 9–11); repeat testing during drug infusion was conducted on the 11th day of either CGRP (*n* = 4; one animal not tested) or CGRP (8–37) (*n* = 7) infusion. RWN recordings were performed in anesthetized animals (20 mg/kg ketamine, 5 mg/kg xylazine) maintained on a warm heating pad. RWN was measured using the round window recording electrode. Using a digital oscilloscope, fast Fourier transformation of ensemble activity was conducted as in Dolan et al. (1990). The broad spectral peak (0.8–1.0 kHz) that is typical of round-window noise was measured. RWN was measured using the implanted electrode; thus, testing was unilateral.

#### DPOAE

Baseline DPOAE tests were conducted once during the second week of unilateral AP infusion (days 9–11); repeat testing during CGRP infusion was conducted on the 9th day of CGRP (*n* = 4; one animal not tested) or CGRP (8–37) (*n* = 5; two animals not tested). Animals were anesthetized (58.8 mg/kg ketamine, 2.4 mg/kg xylazine, and 1.2 mg/kg acepromazine), and placed on a warm heating pad to maintain body temperature, then time-dependent amplitude of the cubic DPOAE (2F_2_-F_1_) was assessed using procedures we have described in detail previously (Halsey et al., 2005; Le Prell et al., 2005). In brief, the DPOAE tests were conducted using TDT system II/III equipment in combination with an Etymotic Research microphone-earphone assembly (ER-10B + Low Noise Microphone) coupled to the subject’s ear by a short segment of flexible vinyl tubing, minimally smaller than the guinea pig ear canal and which sealed the ear canal to provide a closed field test condition. Testing was unilateral and limited to the experimental ear.

Onset adaptation of the DPOAE is a measure of the strength of the MOC reflex (see Liberman et al., 1996; Maison and Liberman, 2000; Kujawa and Liberman, 2001). Onset adaptation of the cubic distortion product was measured as described previously (Halsey et al., 2005; Le Prell et al., 2005); primary tone frequencies (F_1_, F_2_) were fixed at 8 kHz (F_1_) and 9.6 kHz (F_2_) and the cubic distortion product (2F_1_-F_2_) was 6.4 kHz. F_1_ and F_2_ levels (L_1_, L_2_) were initially set to approximately 92-dB SPL; L_2_ was then systematically decreased over a 12-dB range in 1-dB steps. This procedure was repeated for at least six levels of F_1_, with F_1_ decreasing in 1-dB steps, until the levels producing maximum DPOAE adaptation were determined for both positive and negative deflections. Additional tests were then conducted with L_1_ and L_2_ level increments changing in 0.4-dB increments over at least six levels of F_1_, with twelve F_2_ levels presented for each level of F_1_. A MatLab program was used to control stimulus generation (TDT hardware) and presentation (Beyer sound drivers), as well as data collection.

Primary tones were 1 s in duration, with a 1.5 s pause between presentations. Sound levels for F_1_, F_2_, and the DPOAE were determined during each level series using Fourier transform of the microphone input. For each level combination, responses to four stimulus presentations were collected and averaged. DPOAE amplitude was sampled at 50-msec intervals during the 1-s primary tone duration. If standard deviations exceeded 2-dB at any time point, the data were excluded and the level combination was repeated. Adaptation of the DPOAE response was defined as the difference between DPOAE amplitude at the onset of the primary tones and the steady-state amplitude of the DPOAE (defined as the average DPOAE amplitude during the final four time points).

#### Statistical Analysis

All data values in the text and figures are mean ± S.E.M.; all statistical comparisons were performed using SPSS 13.0. Statistical reliability of group differences was via ANOVA. Adjustment for violation of sphericity was accomplished using the Greenhouse-Geisser correction. Bonferroni corrections were applied for multiple comparisons.

CAP data collected during unilateral AP infusion were evaluated as a function of time post-implant (AP1: days 3–8; AP2: days 12–14) to assess possible post-operative changes in function. Functional data collected during unilateral CGRP infusion were evaluated as a function of the duration of drug delivery (CGRP1: days 2–5; CGRP2: days 6–8; CGRP3: days 13–14) to account for increasing intra-cochlear CGRP concentration during the 14-day infusion period.

## Results

### Changes in Evoked Potentials During AP Infusion

Threshold changes during AP were minimal, consistent with Brown et al. (1993) and Le Prell et al. (2004). Whereas Brown et al. (1993) monitored ABR threshold measures in saline-infused animals as a surgical control during the development of this surgical procedure, Le Prell et al. (2004) measured both ABR thresholds and behavioral thresholds during 14–56 days of AP infusion prior to drug manipulation (Le Prell et al., 2004). With respect to CAP amplitude, there was a statistically significant Frequency x Replication (AP1, AP2) effect (*p* = 0.042) of time post-implant on CAP amplitude. Pair-wise comparisons revealed that differences between AP1 and AP2 measurements were limited to a small but statistically reliable decrease in CAP amplitude at 4 (*p* = 0.037) and 8 (*p* = 0.045) kHz, with no time-related changes in CAP amplitude at 16 or 24 kHz (*p*’s > 0.05). Larger decreases in amplitude were evident in the surgical controls included by Sly et al. (2012). They reported small but largely reversible post-surgical reductions in DPOAE amplitude and slowly progressive decreases in ABR wave III amplitude over 4-week of infusion of Ringer’s solution which they attributed to surgical trauma to the cochlea. Effects of CGRP were measured relative to the AP2 baseline collected during days 12–14 of AP infusion.

### Intra-Cochlear CGRP Improved Threshold Sensitivity

**FIGURE 1:**
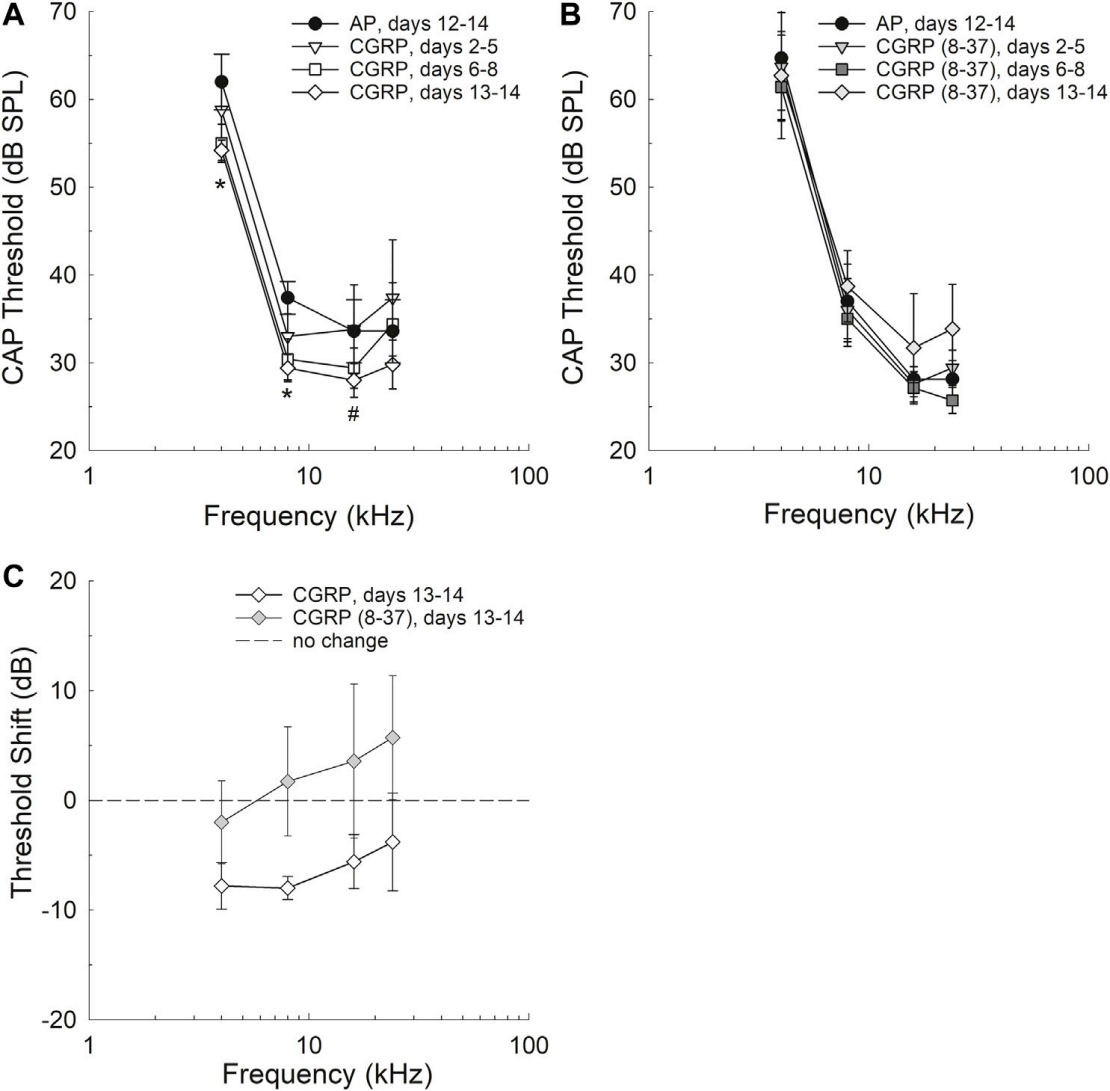
Threshold sensitivity was improved after 13–14 days of CGRP agonist infusion, but not at earlier infusion times including days 2–5 and 6–8 **(A)**. Differences between AP and CGRP thresholds were statistically significant at 4 and 8 kHz (see asterisks) and approached statistical significance at 16 kHz (*p* = 0.0853). Threshold sensitivity after 13–14 days of CGRP antagonist [CGRP (8–37)] infusion was not reliably different from AP control **(B)** and no pair-wise comparisons were completed. The change in thresholds as a function of drug was significantly different when threshold shift as a function of drug assignment was compared **(C)**.

Threshold sensitivity during AP, CGRP, and CGRP (8–37) antagonist infusion are shown in Figures 1A,B; because each subject contributed both baseline (AP) and either CGRP or CGRP (8–37) data points, the within-subject change in threshold is also shown (Figure 1C). CAP threshold was lower (better) during CGRP infusion when compared to within-subject pre-drug (AP) baseline (*p* = 0.043); after Bonferroni correction, the only pair-wise comparison that approached statistical reliability was AP versus the latter CGRP time point (days 13–14, see Figure 1A) (*F*_1,4_ = 6.998, *p* = 0.057). To determine which frequencies had reliable differences between AP and CGRP days 13–14 thresholds, two-tailed paired t-tests were completed at each frequency. Differences were statistically significant at 4 kHz (*t*_4_ = 3.661, *p* = 0.0216) and 8 kHz (t_4_ = 7.682, *p* = 0.00159), and approached statistical significance at 16 kHz (t_4_ = 2.275*p* = 0.0853), but not at 24 kHz (t_4_ = 0.851, *p* = 0.443). CAP threshold measured during infusion of the CGRP receptor antagonist, CGRP (8–37), was not different from baseline (1B) (*F*_1,6_ = 0.187, *p* = 0.681) and no additional pair-wise comparisons were completed within test frequencies. When change in threshold (on day 13–14 of infusion) was compared as a function of the treatment agent [CGRP vs. CGRP (8–37)] using a two-way ANOVA with treatment and frequency as factors, there was a statistically significant difference between the treatment groups (*F*_1,47_ = 6.003, *p =* 0.019) with no effect of frequency (*F*_3,47_ = 0.532, *p* = 0.663) and no drug × frequency interaction (*F*_3,47_ = 0.0700, *p* = 0.976).

### Intra-Cochlear CGRP Enhanced CAP Amplitude

**FIGURE 2:**
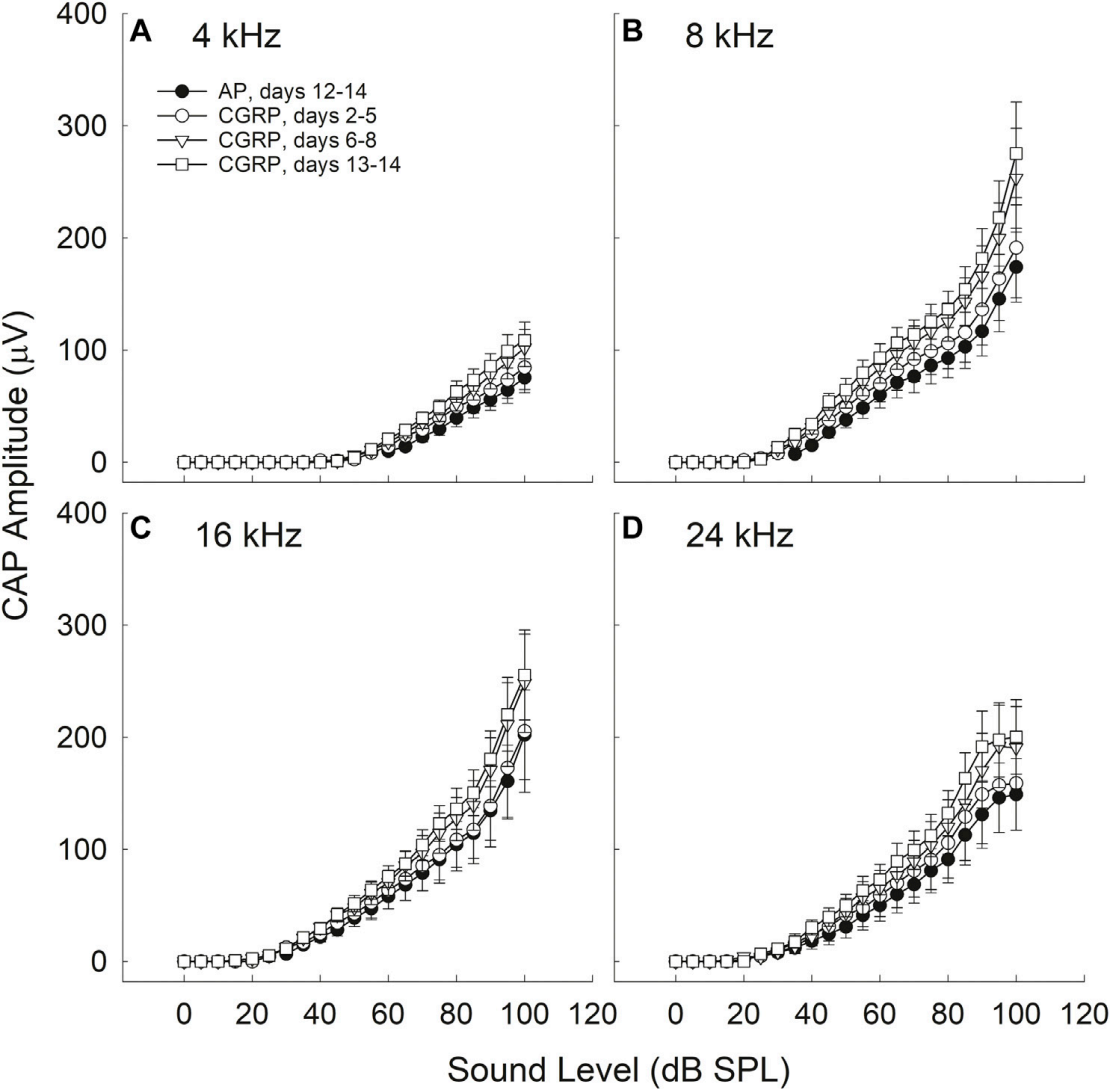
CAP amplitude increased during CGRP infusion; all comparisons are relative to control measurements on the 12th-14th day of artificial perilymph (AP) infusion, immediately prior to the pump change surgery during which the AP pump was replaced with a pump containing CGRP. Increases in CAP amplitude (30–35%) were statistically reliable at 4 **(A)**, 8 **(B)**, 16 **(C)**, and 24 **(D)** kHz for the CGRP (days 13–14) comparisons with the AP data set (all *p*‘s < 0.005). CGRP-induced changes are shown as a function of duration of infusion; tests were conducted at least once from days 2–5, on days 6, 7, or 8, and on days 13 or 14.

CGRP-induced increases in CAP amplitude were time-, frequency-, and intensity-dependent (see Figure 2).

#### Time-Dependence

There were no reliable CGRP-induced changes in CAP amplitude during the earliest infusion days (days 2–4). When CAP amplitude was assessed on days 6–8 of infusion, the increase in CAP amplitude (20–30%) approached statistical reliability (4 kHz, *p* = 0.055; 8 kHz, *p* = 0.071; 16 kHz; *p* = 0.136; 24 kHz, *p* = 0.053). The most robust increases in CAP amplitude (30–35%) were observed at the 13–14 days time point, and these amplitude increases were statistically reliable (4 kHz, *p* = 0.016; 8 kHz, *p* = 0.026; 16 kHz, *p* = 0.052; 24 kHz, *p* = 0.027). Time-dependent changes are likely a consequence of the slow infusion rate of the osmotic mini-pump, with significant drug accumulations building slowly over multiple days or weeks (for discussion see Le Prell et al., 2004).

#### Frequency-Dependence

For the data set collected on the 13th and 14th days of infusion, a time at which the effects of CGRP on CAP amplitude were highly reliable, there was a statistically significant Condition × Frequency interaction (*p* = 0.022). The difference between AP and CGRP was smaller at 4 kHz than at the other (higher) test frequencies. Smaller effects at the lowest frequencies, furthest from the site of infusion, are not surprising as drug diffusion depends on the slow cochlear fluid gradient (approximately 1.6 nl/min, see Ohyama et al., 1988; for discussion see Le Prell et al., 2004). LOC innervation has been sparser in the apex than in the base in some (Safieddine and Eybalin, 1992) but not all (Sliwinska-Kowalska et al., 1989; Ylikoski et al., 1989) reports thus frequency effects could be related not only to drug distribution but also to CGRP receptor distribution.

#### Intensity-dependence

**FIGURE 3:**
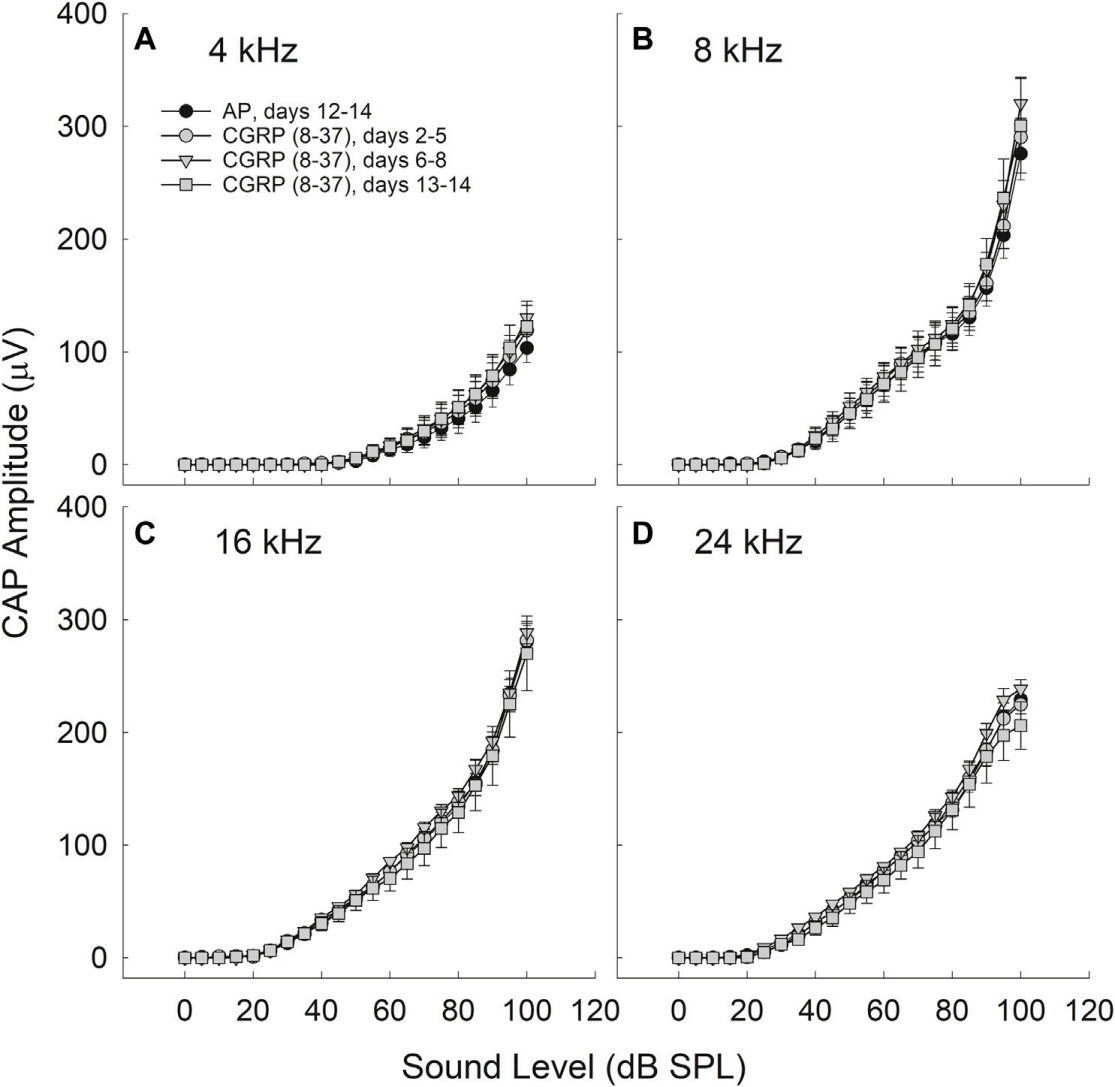
CAP amplitude did not change as a function of CGRP (8–37) infusion at 4 **(A)**, 8 **(B)**, 16 **(C)**, and 24 **(D)** kHz. CAP tests were conducted at least once from days 2–5, on days 6, 7, or 8, and on days 13 or 14 for CGRP (8–37). All comparisons are relative to control measurements on the 12th–14th day of artificial perilymph (AP) infusion, immediately prior to the pump change surgery during which the AP pump was replaced with a pump containing CGRP (8–37).

The difference in CAP amplitude during AP and CGRP increased with increasing intensity (4 kHz: *p* = 0.028; 8 kHz: *p* = 0.077; 16 kHz: *p* = 0.069; 24 kHz: *p* = 0.019). In contrast to the statistically significant time, frequency, and intensity dependent effects of CGRP infusion, there were no statistically significant treatment effects for CGRP (8–37) (*F*_1,6_ = 0.004, *p* = 0.984) (Figure 3).

### Intra-Cochlear CGRP Increased Spontaneous Neural Activity

**FIGURE 4:**
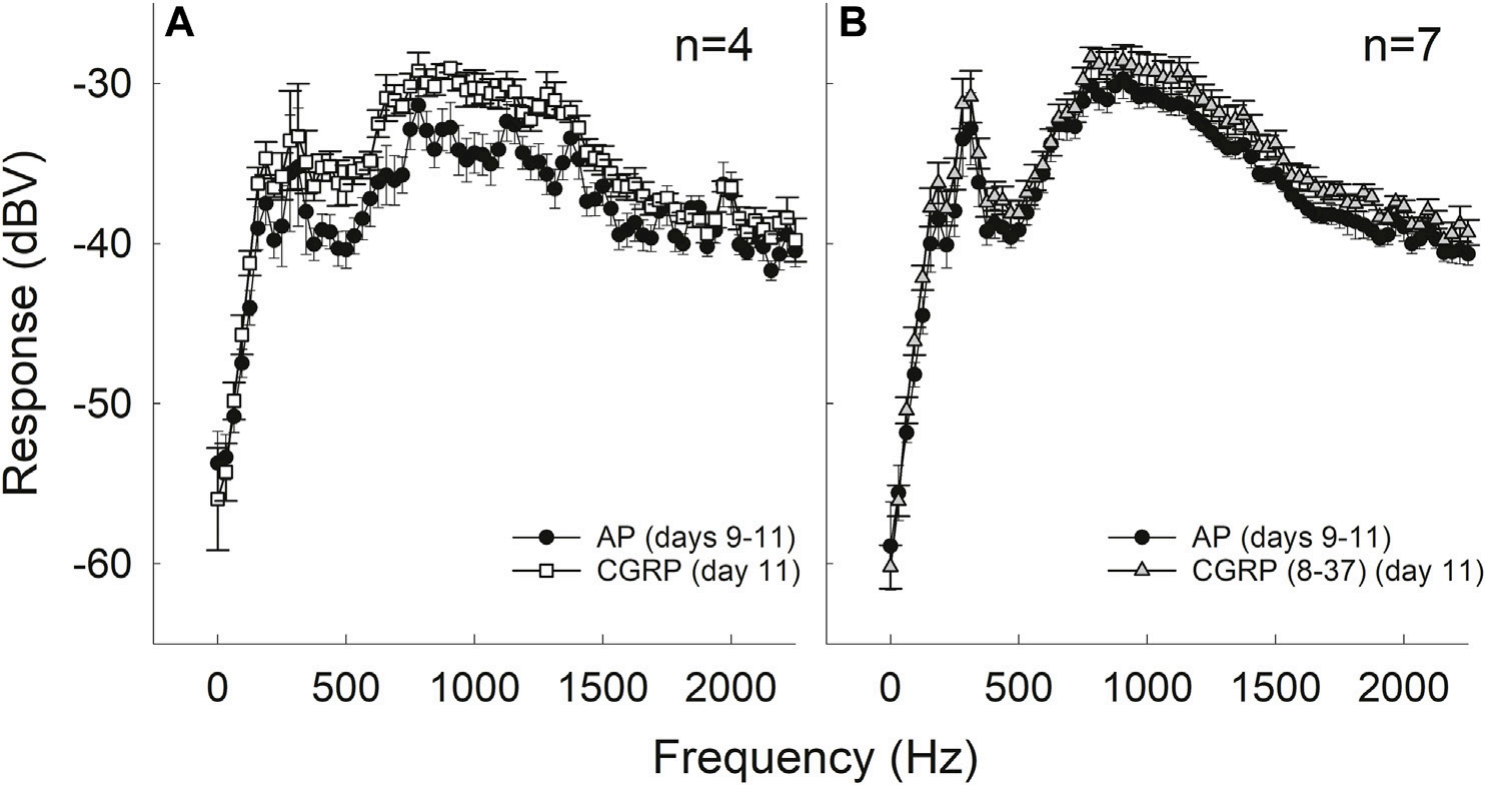
Intra-cochlear infusion of CGRP enhanced round window noise from approximately 500–1,200 Hz; the frequency range associated with spontaneous firing of the auditory nerve **(A)**. There were no changes in round window noise during infusion of CGRP (8–37) **(B)**.

Electrical activity at the round window increased during CGRP infusion in the 800–1,200 Hz region (*F*_1,3_ = 13.025, *p* = 0.037); this “hump” region reflects spontaneous firing of multiple auditory nerve fibers (Figure 4A) (for discussion of ‘hump’ region, see Kiang et al., 1976; Dolan et al., 1990; Groff and Liberman, 2003). In contrast, comparisons between AP and the antagonist, CGRP (8–37), revealed no significant differences in electrical activity (*F*_1,6_ = 3.229, *p* = 0.122) (Figure 4B). Baseline measures in the group of guinea pigs that later received CGRP systematically differed from the baseline measures in the group that later received CGRP (8–37) (*F*_1,9_ = 4.830, *p* = 0.056 when all frequencies were used and *F*_1,9_ = 5.625, *p* = 0.042 when only frequencies less than 1,100 Hz were used).

### Intra-Cochlear CGRP Does not Affect MOC Reflex Strength

**FIGURE 5:**
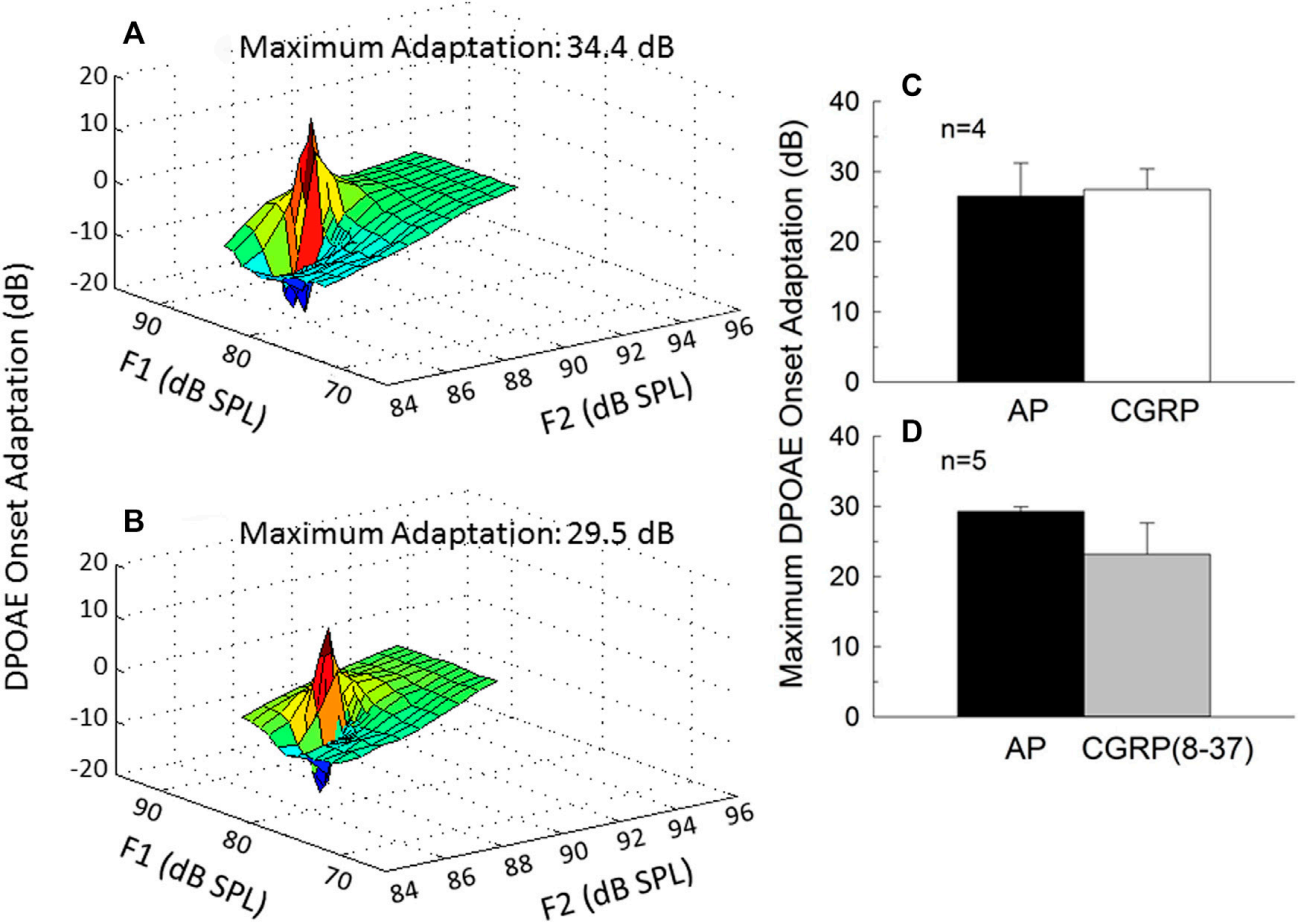
Onset adaptation of the outer hair cell distortion product otoacoustic emission (DPOAE) shows a rapid level-dependent adaptation shortly after signal onset when the medial olivocochlear pathway is intact. DPOAE adaptation is depicted on day 9 of artificial perilymph **(A)** and CGRP **(B)** infusion for one representative animal. The maximum adaptation was extracted from the fine-structure plots shown in **(A,B)**. Onset adaptation was not reliably affected by either CGRP **(C)** or CGRP (8–37) **(D)** (all *p*’s > 0.25).

The strength of the MOC reflex, measured using onset adaptation of the DPOAE, was essentially the same during AP, CGRP infusion, and CGRP (8–37). DPOAE adaptation is illustrated for one representative animal in Figure 5 (5A: AP; 5B: CGRP). Adaptation was clearly robust (>20 dB) in both conditions; mean adaptation data for all animals is illustrated in Figure 5 [5C: CGRP; 5D: CGRP (8–37)]. Although adaptation magnitude was seemingly reduced during infusion of the CGRP receptor antagonist, these differences were not statistically reliable (all *p*’s > 0.25). In contrast to the lack of effect shown here, manipulations known to disrupt the MOC system, including intra-venous strychnine and OCB transection, cause a profound depression of DPOAE adaptation (approximately 20-dB, see Le Prell et al., 2005).

### Intra-Cochlear CGRP Does not Affect Outer Hair Cell Function

**FIGURE 6:**
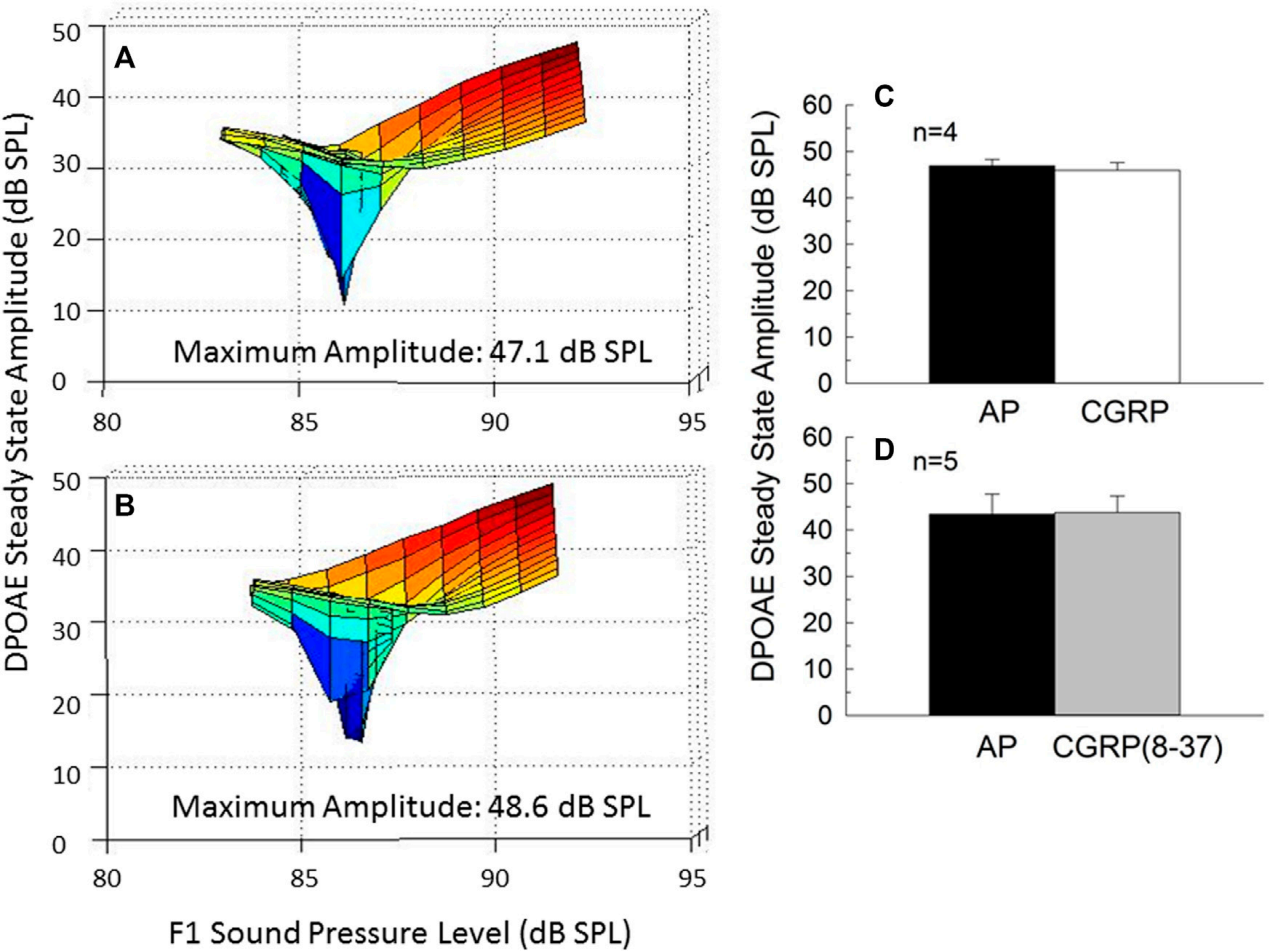
Steady-state amplitude of the outer hair cell distortion product otoacoustic emission (DPOAE) varies with F1 and F2 sound level, and provides as a measure of the integrity of the outer hair cell population. DPOAE amplitude is depicted on day 9 of artificial perilymph **(A)** and CGRP **(B)** infusion for one representative animal. The steady state amplitude was extracted from the fine-structure plots shown in **(A,B)**. The level-dependent steady-state DPOAE amplitude was unchanged by either CGRP **(C)** or CGRP (8–37) **(D)** infusion (all *p*’s > 0.05).

DPOAE amplitude, a measure of the functional integrity of the OHC population, is illustrated for one representative animal in Figure 6 (6A: AP; 6B: CGRP). Mean steady-state amplitude data for all animals is illustrated in Figure 6 [6C: CGRP; 6D: CGRP (8–37)]. The lack of effect of CGRP and CGRP (8–37) on DPOAE amplitude indicates that OHC function was not influenced by intra-cochlear infusion of CGRP ligands, at least within the range of levels tested here. Lack of effect of CGRP and CGRP (8–37) contrasts with profound depression of DPOAE steady-state amplitude following intra-cochlear neomycin (Le Prell et al., 2004) or euthanasia with an overdose of sodium pentobarbital (Le Prell et al., 2005).

## Discussion

The primary outcome reported here is an increase in CAP amplitude accompanied by small improvements in CAP threshold during CGRP infusion into the cochlea. These results are consistent with the decreases in ABR Wave I amplitude reported by Maison et al. (2003b), who observed a decrease in sound-driven auditory nerve activity in the absence of endogenous αCGRP. Although no changes in thresholds were detected in the 16-week old mutant mice described by Maison et al. (2003b), changes in thresholds were detected in the 3-month old mutant mice described by Allen and Luebke (2017), with CGRP-null mice having poorer hearing in both quiet and in noise. The current results in guinea pigs are consistent with Allen and Luebke (2017) in that thresholds were better when exogenous CGRP was infused; however, threshold deficits did not emerge when CGRP (8–37) was infused. In addition to sound-evoked neural response, the current study evaluated spontaneous neural activity using round window noise. The increased round window noise described here during CGRP infusion suggests that CGRP induced an increase in spontaneous AN firing. The observed effects are consistent not only with data from CGRP-null mice, but also with the increase in spontaneous activity observed after bath application of CGRP to *Xenopus* lateral line organ (Adams et al., 1987; Sewell and Starr, 1991; Bailey and Swell, 2000a; Bailey and Swell, 2000b). Having demonstrated excitatory effects of CGRP in the current study, additional studies are warranted to determine a dose-response curve. A preliminary test with a single subject infused with a lower dose (125 μM) showed no effects of CGRP on CAP threshold or amplitude at any of the test times over the 14-day infusion, but the lowest effective dose is not known, and the effects of increasing either the concentration of CGRP or the duration of CGRP infusion are not known.

In their description of the effects of CGRP-null mutations, Maison et al. (2003b) reported they did not observe any changes in DPOAE threshold or amplitude, or the strength of the MOC reflex. Allen and Luebke (2017) similarly reported DPOAE responses to be intact in CGRP-null mice. The lack of effect of CGRP-null mutations on measures of OHC function is perhaps surprising given robust CGRP-positive immunolabeling of the MOC efferents that innervate the OHCs (Cabanillas and Luebke, 2002; Kong et al., 2002; Maison et al., 2003a). However, the data collected here using both CGRP and the CGRP (8–37) fragment, a receptor antagonist, were consistent with the reports by Maison et al. (2003b) and Allen and Luebke (2017). Just as CGRP-null mice did not have any changes with respect to DPOAE metrics, no changes were observed here in guinea pigs that underwent chemical manipulation of the cochlea fluids with CGRP ligands. Taken together, the data show CGRP effects on the AN but not the OHCs, although we cannot exclude the possibility for as-yet unknown effects of CGRP on the OHCs. Future studies should continue to include detailed input-output functions for DPOAE responses across a wide range of sound levels to assure that OHC function is well characterized.

The earlier data from Maison et al. (2003b) and Allen and Luebke (2017) reflect the loss of CGRP-mediated modulation of AN activity that is present in the wild-type controls. In mutant CGRP-null mice there is a loss of excitation; here, with CGRP infusion, there is evidence of increased spontaneous and sound driven AN activity. While we interpret these data to reflect direct effects of CGRP on the AN, the possibility that CGRP-mediated changes in blood flow could also contribute to the observed results must be considered. The spiral modiolar artery, a primary supplier of blood to the cochlea, contains CGRP-positive terminals (Ylikoski et al., 1989; Carlisle et al., 1990; Lyon and Payman, 2000; Qiu et al., 2001; Herzog et al., 2002) as well as CLR mRNA (Herzog et al., 2002). Micro-infusion of CGRP directly into the arterial network of the cochlea increases cochlear blood flow (Quirk et al., 1994), as does intra-venous CGRP infusion (Hillerdal and Andersson, 1991). However, when drugs that influence cochlear blood flow have been delivered directly into the cochlear fluids via round window application in the rat, instead of being delivered directly into the bloodstream, the otherwise vasoactive drugs failed to reduce cochlear blood flow (Laurikainen et al., 1993). Because the CGRP ligands were administered into the cochlear fluids in this study, the data from Laurikainen et al. (1993) suggest the current infusions to be unlikely to influence cochlear blood flow. However, even if the CGRP ligands did move into the blood supply, intra-arterial CGRP has no effect on CAP (Quirk et al., 1994), and other blood-flow promoting drugs have also failed to affect CAP amplitude after intra-venous drug delivery (Lamm and Arnold, 2000).

Changes in CAP have accompanied cochlear blood flow alterations only if the drug treatment resulted in arterial blockade, ischemia, and excitotoxic AN swelling, in which case CAP decreases (e.g., Asai et al., 1996; Kong et al., 1996; Scheibe et al., 1997; Tabuchi et al., 1998). Thus, even if intra-cochlear CGRP did influence cochlear blood flow, the observed increases in CAP amplitude are not readily explained by changes in cochlear blood flow. It is possible that inflammation affecting the cochlear vascular system could occur after cochlear surgery, and inflammation has the potential to influence cochlear blood flow (Sharaf et al., 2016). However, normal post-operative hearing thresholds suggest inflammation not to be a major concern as inflammation is increasingly understood to be associated with hearing loss (Frye et al., 2019; Zhang et al., 2021). Here, thresholds were not only normal, they showed statistically significant decreases (improvements) during CGRP infusion, which would not be expected in the presence of surgical trauma and significant inflammation. Taken together, the overall pattern of results is more consistent with direct effects on the AN than with indirect effects on the AN mediated via changes in cochlear blood flow.

Previous studies have showed that cutting the olivocochlear bundle (OCB), which eliminates both MOC and LOC innervation, depressed spontaneous (Liberman, 1990; Walsh et al., 1998; Zheng et al., 1999) and sound-driven (Zheng et al., 1999) AN activity. When the LSO is lesioned in the guinea pig brainstem using melittin (Le Prell et al., 2003b) or the LOC system is lesioned in the guinea pig cochlea using MPTP (Le Prell et al., 2005), sound-driven AN activity is decreased. When the guinea pig LOC system is lesioned using MPTP, spontaneous AN firing is also decreased (Le Prell et al., 2014a). We have long speculated that the decreases in spontaneous and sound driven AN firing after OCB transection, LSO lesion, or LOC lesion could be the result of compromised CGRP release when the LOC system is disrupted (Le Prell et al., 2003b; Le Prell et al., 2005; Le Prell, 2007; Le Prell et al., 2014a). The observation that CGRP infusion directly increased ensemble spontaneous and sound-driven AN activity in this study adds new support for the hypothesis that endogenous CGRP release by the LOC efferent neurons has an excitatory effect on the AN.

The chemical transmitters that might mediate the net-excitatory effect in the intact (undamaged) system are a question of significant interest (for review and discussion see Le Prell et al., 2003a; Le Prell et al., 2003b; Le Prell et al., 2005; Reijntjes and Pyott, 2016). In brief, most data suggest that DA is largely inhibitory with respect to AN activity (d’Aldin et al., 1995a; d’Aldin et al., 1995b; Oestreicher et al., 1997; Ruel et al., 2001), although new data using selective agonists and antagonists acting at D_2_ receptors suggest tonic release of endogenous DA has an excitatory action at the D_2_ receptor (Garrett et al., 2011). Similar, but more limited, documentation of inhibitory effects on AN activity have been reported for enk (Burki et al., 1993) and GABA (Felix and Ehrenberger, 1992; Arnold et al., 1998). Comprehensive discussion of these diverse pharmacological studies is available in Reijntjes and Pyott (2016). Recent data suggest differential release of ACh and DA in different sound conditions and a “fine-tuning” of the activity of the auditory nerve via the release of these two LOC neurotransmitters (Wu et al., 2020).

Overall understanding of the LOC system will require improved understanding of other LOC transmitters that are co-localized with CGRP in the LOC terminals, including, for example, ACh and dyn. The effects of ACh on the OHCs have been carefully characterized, although the potential effects of ACh on the AN remain less well understood (for detailed reviews, see Puel, 1995; Le Prell et al., 2001; Reijntjes and Pyott, 2016; Fuchs and Lauer, 2019). Some studies have used cochlear perfusion, and others have used microiontophoresis to restrict ACh delivery to the vicinity of the AN dendrites; chemical antagonists and genetic manipulations have also been employed. Release of ACh from the MOC efferents onto the OHCs inhibits OHC electromotile action. This body of work suggests that the α9/α10 nicotinic receptors may provide a novel pharmacotherapeutic target (Elgoyhen et al., 2009). Early data collected with ACh delivered via intracochlear perfusion indicated that 250 μM ACh had no effect on the cochlear microphonic (CM) or CAP; however, 250 μM ACh delivered in combination with 10 μM eserine (which blocks the metabolism of ACh) augmented CM amplitude and depressed CAP amplitude, suggesting neural effects may be secondary to changes in OHC response to sound (Bobbin and Konishi, 1971). In contrast to the decreases in CAP with cochlear perfusion, when 0.5 M ACh was applied in the vicinity of the AN dendrites using microiontophoresis, increased subsynaptic spiking and enhanced glutamate-induced AN activity were observed, suggesting direct excitatory effects of ACh on the AN (Felix and Ehrenberger, 1992). Other recent data also suggest direct excitatory effects of ACh on the AN; specifically, selective deletion of several muscarinic receptor (M_1_ and/or M_4_) subtypes decreased neural response amplitude without effecting DPOAEs (Maison et al., 2010). Taken together, this data suggests the potential that ACh may also serve as an excitatory transmitter when released from the LOC efferent neurons.

The effects of dynorphin (dyn) have been less clear, with data from Sahley and colleagues, who assessed supra-physiological doses, suggesting excitatory (Sahley et al., 1991; Sahley and Nodar, 1994), or biphasic (Sahley et al., 2008) effects. In contrast, data collected using biologically plausible drug concentrations instead suggested that dyn inhibits sound-evoked AN activity (Le Prell et al., 2014b). More recently, Sahley et al. (2019) suggested that release of dyn by the LOC neurons may in part drive well-observed cascades including glutamate-induced swelling, inflammation, excitotoxicity, and hearing loss as dyn has been linked to such responses (immune, inflammatory, and excitotoxic) in other neural systems. Taken together, the transmitter data collected using DA, GABA, enk, and dyn have largely failed to explain the net excitatory effects of LOC innervation on the AN that are suggested by the net-inhibitory effects of LOC lesions, as well as observation that electrical current applied at some locations within the LSO enhances CAP amplitude (Groff and Liberman, 2003). The data from Maison et al. (2003b) and Allen and Luebke (2017) using CGRP-null mice suggest CGRP as a candidate transmitter substance, and the current data using chemical manipulations confirm CGRP has the potential to upregulate AN activity, with ACh also remaining a potentially excitatory neurotransmitter substance within the LOC system.

The extent to which endogenous CGRP is released from the LOC efferents in either a tonic state or stimulus-driven (sound) condition is not currently known; thus, important questions remain. In an effort to identify endogenous release, we assessed the effects of a CGRP receptor antagonist, CGRP (8–37). The finding that CGRP (8–37) did not influence AN or OHC outcomes may suggest there is no appreciable tonic or sound-driven release of CGRP by the LOC or MOC efferents, at least under these test conditions. Caution is required with respect to this interpretation however, as the extent to which CGRP (8–37) is an effective antagonist in the guinea pig inner ear remains an open question. It would be worthwhile to include combinations of agents in future studies to directly assess whether the antagonist selected for use competitively blocks the effects attributed to agonist infusion, and we strongly encourage this approach in future investigations to reduce questions about the potential effectiveness of the antagonist selected for investigation. Another approach that might be used to more precisely localize the observed drug effects to the LOC system would be repeating the drug studies in guinea pigs with efferent lesions, although LOC lesions are often incomplete which would make data from such studies difficult to interpret. LSO lesions induced in the brainstem using melittin do not completely eliminate the LOC innervation in the cochlea (Le Prell et al., 2003b), and LOC lesions induced in the cochlea using MPTP are also incomplete (Le Prell et al., 2005).

We selected CGRP (8–37) as competitive blockade of CGRP1 receptors has been shown in guinea pig basilar artery using CGRP (8–37) (Jansen-Olesen et al., 2001). In addition, CGRP (8–37) has been an effective antagonist when delivered in equimolar concentrations with CGRP (Buffelli et al., 2001). From a practical perspective, CGRP (8–37) was the only antagonist readily available through common commercial sources. However, CGRP (8–37) is widely regarded to not be very potent as an antagonist (Doods et al., 2000), and there have been significant efforts to develop more potent antagonists. The novel CGRP receptor antagonist BIBN4096BS (olcegepant) has been an effective antagonist when applied to brainstem slices from the mouse (Zheng et al., 2021), resulted in decreased bone volume when administered systematically in the mouse (Köhli et al., 2021), and decreased CGRP-induced light aversion when olcegepant was co-administered in the mouse (Russo et al., 2009). In contrast, neither olcegepant nor CGRP (8–37) affected uptake of CGRP in rat dura mater preparations (Gupta et al., 2010). Olcegepant has not been well studied in guinea pigs and thus it is not clear if olcegepant would be more effective than CGRP (8–37) when small drug volumes are infused into guinea pig cochlea. However, olcegepant binding in rat, rabbit, dog, and guinea pig models has been reported to be is as much as 100 times less than that observed in primate models (rhesus macaque, marmoset) (Doods et al., 2000; Hershey et al., 2005).

Given better binding in non-human primates and in humans, multiple drugs that antagonize CGRP release or the CGRP receptor are either in clinical trials or approved for use for antimigraine purposes (Mohanty and Lippmann, 2020). Despite the availability of drugs that are potent in non-human primates and humans, there continues to be a need for antagonists that are effective in rodent models for use in basic (pre-clinical) investigation. In addition to binding issues, stability of the antagonists must be considered for chronic infusion paradigms such as this one. The stability of CGRP (8–37) *in vivo* has been questioned (Miranda et al., 2013). However, CGRP (8–37) was biologically effective when delivered via osmotic mini-pump for 7-days *in vivo* in rats (Reinshagen et al., 1998; Buffelli et al., 2001; Yin et al., 2004). Our infusions extended over a longer period, to 14 days. However, even if CGRP (8–37) were inactivated at later test times, we did not observe any functional changes within the first 6–8 days (see Figure 3), times at which the data from rats suggest the CGRP (8–37) fragment to be stable via osmotic mini-pump delivery.

In summary, this is the first direct demonstration that CGRP infusion enhances spontaneous and sound-driven AN activity. Based on these results, we conclude that CGRP has the potential to play an excitatory role in the AN response, confirming and extending data from knock-out mice (see Maison et al., 2003b). These key data set the stage for psychophysical data to be collected during periods of chronic cochlear drug infusion using osmotic mini-pumps (as in Le Prell et al., 2004) to confirm the possible role of CGRP in modulation of hearing in noise, following the decreased hearing-in-noise documented in CGRP-null mice by Allen and Luebke (2017). Studies in awake animals would have the added benefit of precluding the possibility that ketamine anesthesia may attenuate responses to CGRP. The effects of CGRP are commonly measured in ketamine-anesthetized animals (Radziszewski et al., 2003; Sakamoto et al., 2014; Sakamoto et al., 2017; Søndergaard et al., 2019). However, intra-venous ketamine attenuated CGRP-induced vasodilation of the dural artery in rats (Chan et al., 2010). Possible effects of ketamine on the excitatory effects of CGRP in the cochlea are speculative; however, if CGRP has less excitatory effect when measured under ketamine anesthesia, then the effect of CGRP on AN activity could be even more robust than was shown in this report. Taken together, the data provide direct confirmation of excitatory effects of CGRP on the AN, and support a potentially important role for CGRP in auditory function.

## Data Availability

The raw data supporting the conclusion of this article will be made available by the authors, without undue reservation.
